# Association between metabolically healthy obesity and risk of atrial fibrillation: taking physical activity into consideration

**DOI:** 10.1186/s12933-022-01644-z

**Published:** 2022-10-13

**Authors:** Ruoting Wang, Ivan Olier, Sandra Ortega-Martorell, Yingxin Liu, Zebing Ye, Gregory YH Lip, Guowei Li

**Affiliations:** 1 Center for Clinical Epidemiology and Methodology (CCEM), Guangdong Second Provincial General Hospital, Guangzhou, China; 2 Liverpool Centre for Cardiovascular Science, University of Liverpool and Liverpool Heart & Chest Hospital, Liverpool, UK; 3 School of Computer Science and Mathematics, Liverpool John Moores University, Liverpool, UK; 4 Department of Cardiology, Guangdong Second Provincial General Hospital, Guangzhou, China; 5 Aalborg Thrombosis Research Unit, Department of Clinical Medicine, Aalborg University, Aalborg, Denmark; 6 Department of Health Research Methods, Evidence, and Impact, McMaster University, Hamilton, Canada; 7 CCEM, Guangdong Second Provincial General Hospital, 510317 Guangzhou, China; 8 Department of HEI, McMaster University, 1280 Main St West, L8S 4L8 Hamilton, ON Canada

**Keywords:** Atrial fibrillation, Obesity, Metabolic status, Physical activity

## Abstract

The modification of physical activity (PA) on the metabolic status in relation to atrial fibrillation (AF) in obesity remains unknown. We aimed to investigate the independent and joint associations of metabolic status and PA with the risk of AF in obese population. Based on the data from UK Biobank study, we used Cox proportional hazards models for analyses. Metabolic status was categorized into metabolically healthy obesity (MHO) and metabolically unhealthy obesity (MUO). PA was categorized into four groups according to the level of moderate-to-vigorous PA (MVPA): none, low, medium, and high. A total of 119,424 obese participants were included for analyses. MHO was significantly associated with a 35% reduced AF risk compared with MUO (HR = 0.65, 95% CI: 0.57–0.73). No significant modification of PA on AF risk among individuals with MHO was found. Among the MUO participants, individuals with medium and high PA had significantly lower AF risk compared with no MVPA (HR = 0.84, 95% CI: 0.74–0.95, and HR = 0.87, 95% CI: 0.78–0.96 for medium and high PA, respectively). As the severity of MUO increased, the modification of PA on AF risk was elevated accordingly. To conclude, MHO was significantly associated with a reduced risk of AF when compared with MUO in obese participants. PA could significantly modify the relationship between metabolic status and risk of AF among MUO participants, with particular benefits of PA associated with the reduced AF risk as the MUO severity elevated.

**Supplementary information:**

The online version contains supplementary material available at 10.1186/s12933-022-01644-z.

## Introduction

Atrial fibrillation (AF) is a major cause of morbidity and mortality worldwide, including a five-fold increased risk of stroke and a substantial healthcare burden [1–3]. There were a large number of AF cases undetected and untreated, posing a severe challenge to stroke prevention especially in the aging population [4]. Therefore, predicting the onset of AF and identifying strategies for AF prevention are of significant importance for the public health. Previous studies have demonstrated that obesity is an important contributing factor for developing AF [5–8]. Based on various metabolic features including blood pressure, glucose tolerance, and lipid profile, obesity can be categorized into two categories: metabolically healthy obesity (MHO) and metabolically unhealthy obesity (MUO) [9–11]. One nationwide study in Asia suggested that metabolically unhealthy patients had significantly higher AF risk when compared with a metabolically healthy population [12]. However, evidence of metabolic status in relation to AF among *obese* individuals remains scarce. Hence, the relationship between metabolic status and AF risk in the obese population requires further exploration [13].

Physical activity (PA) is a major modifiable factor for cardiovascular disease [14–16]. Moreover, some observational studies have suggested a positive association between PA and the risk of AF onset [17–19]. Nevertheless, previous reports did not consider the modification of PA on the association between metabolic status and AF risk. This is important given the focus on integrated care management of AF in contemporary guidelines, including attention to comorbidities and lifestyle factors [20–22].

In this study, we aimed to investigate the independent and joint associations of metabolic status and PA with the risk of AF in obese population using the data from the nationwide United Kingdom (UK) Biobank study.

## Methods

### Study participants

The UK Biobank covers more than 500,000 participants with middle and old age recruited between 2006 and 2010. All participants signed written informed consent, and completed touch-screen questionnaires, interviews with trained medical personnel and physical measurements. Details of the study design and data collection have been previously reported [23].

There were 122,244 obese participants at baseline, among whom 2,820 individuals were excluded because they had AF at baseline or a history of AF. We used the information from self-reported illness and operation history, disease diagnosis codes linkage of international classification of diseases ninth (ICD-9) and 10th (ICD-10) revisions, and operative procedure codes (OPCS) to identify baseline AF (**STable 1**). Overall, a total of 119,424 participants were included for the present analysis (**SFigure 1**). All participants were followed up from baseline until an AF diagnosis, death, or the censoring date (March 2017 for England and October 2016 for Scotland), whichever came first.

### Outcomes

Our primary outcome was incident AF, and the secondary outcome was AF mortality. In the UK Biobank, information on incident AF was evaluated by linkage with hospital in-patient data and death registry records. Specifically, the incident AF was identified by using the combination of disease diagnosis codes linkage of ICD-9 and ICD-10, OPCS, and death registry records during follow-up. We used the death registry records to document the AF mortality, in which the AF was the primary or secondary cause of death. **STable 1** displays the codes used for the identification of incident AF and AF mortality.

### Exposures

The information on exposures was obtained at baseline. ‘Metabolically healthy’ indicated none of the metabolic disorders, and ‘metabolically unhealthy’ indicated at least one of the metabolic disorders, where metabolic disorders included hypertension, hypercholesterolemia, and diabetes [24, 25].

Participants who met at least one of the following criteria were deemed to be hypertensive: systolic blood pressure ≥ 140 mmHg, diastolic blood pressure ≥ 90 mmHg, taking anti-hypertensive medications, a self-reported history of hypertension or ICD-10 code I10-I15 and ICD-9 code 401 and 403. Self-reported history of high cholesterol, taking medications, or ICD-10 code E780 and ICD-9 code 2720 was the criteria for hypercholesterolemia. Diabetes mellitus was defined as hospital records of diabetes at baseline based on ICD-10 code E10-E14 and ICD-9 code 250 or taking medications or a self-reported history of type 1 or type 2 diabetes. The 119,424 obese participants were categorized into MHO and MUO.

PA information, including the frequency and duration of activities, was collected at baseline with the short form International Physical Activity Questionnaire (IPAQ) [26]. PA was presented as metabolic equivalent task-(MET)-mins per week computed by multiplying the MET score by the weekly duration of activity. In line with public health guidelines, PA was categorized into four groups according to the level of moderate-to-vigorous PA (MVPA): none (0 MET-mins per week for MVPA), low (< 600 MET-mins per week), medium (600–1200 MET-mins per week), and high (≥ 1200 MET-mins per week) [26, 27].

### Other independent variables

Covariates of consideration included age (in years), sex (males and females), body mass index (BMI; in kg/m^2^), smoking status (current, previous or never), alcohol drinking status (current, previous or never), employment status (unemployment, retired, employed in night shift work, employed in day shift work, or employed not in shift work), income (< £ 18,000, £ 18,000 - £ 30,999, £ 31,000 - £ 51,999, £ 52,000 - £ 100,000, or > £ 100,000), sleep scores (the number of healthy sleep characteristics: not usual insomnia, no frequent daytime sleepiness, no habitual snoring, morning chronotype, and adequate sleep duration), socioeconomic status (TDI: Townsend deprivation index), vegetable and fruit intake, sedentary behavior (in hours), mental health issues (yes or no) and cardiovascular disease (yes or no) [26, 28, 29].

### Statistical analyses

We performed descriptive analysis for continuous variables (mean and standard deviation [SD]) and categorical variables (counts and percentages). Chi-square test and two independent t-tests were conducted for the baseline characteristics by metabolic status.

Cox proportional hazards models were used to investigate the independent and joint associations of metabolic status and PA with the risk of AF in obese participants. The results were quantified as hazard ratios (HRs) and 95% confidence intervals (CIs). We used MUO, no MVPA, or combination of both as reference. Results of the age- and sex-adjusted model and the fully adjusted model were shown. The latter was obtained after adjusting for age, sex, body mass index, smoking status, alcohol drinking status, employment status, income, sleep scores, socioeconomic status, vegetable and fruit intake, sedentary behavior, mental health issues and cardiovascular disease. We also conducted subgroup analyses and added interaction terms in the model to explore whether there were differences in joint associations of metabolic status and PA with the risk of AF, including sex (males versus females), and age (< 65 versus ≥ 65 years) groups.

In an *exploratory analysis*, we further explored the modification of PA in different metabolic statuses including MHO status and three MUO statuses: mild (with only one of the metabolic disorders), moderate (with two of the metabolic disorders), and severe (with all of three metabolic disorders). Furthermore, we performed additional analyses for modification of metabolic status and PA with AF risk among obese individuals, where metabolic status is the exposure of interest and PA is the potential modifier [30].

We conducted a sensitivity analysis by using multiple imputation techniques for the missing data of PA to assess the joint associations. As another sensitivity analysis, we examined the joint associations by using a different definition of the metabolic status. Participants with ≥ 2 of the following criteria were considered as MUO: (1) systolic blood pressure ≥ 130 mmHg, diastolic blood pressure ≥ 85 mmHg, or taking anti-hypertensive medications, (2) triglycerides ≥ 1.7 mmol/L or taking lipid-lowering drugs, (3) fasting glucose ≥ 5.6 mmol/L or taking medications for diabetes, and (4) HDL-cholesterol < 1.0 mmol/L for male and < 1.3 mmol/L for female [31]. Furthermore, we performed another *post hoc* sensitivity analysis by adjusting for age, sex, height, C-reactive protein, white blood cell counts, smoking status, alcohol drinking status, income, sleep scores, mental health issues, employment status, socioeconomic status, vegetable and fruit intake, sedentary behavior, and cardiovascular disease.

All the statistical analyses were conducted in SAS software version 9.4 and R software version 4.1.1.

## Results

A total of 119,424 obese participants were included for analyses, with a median follow-up of 8.1 years. The description of participants’ baseline characteristics by metabolic status was summarized in Table 1. Participants with MUO (77%) were older, less likely to be physically active, poorer, and more sedentary compared with MHO participants. A significantly higher BMI was found in participants with MUO. There were more males, smokers in participants with MUO, while MHO individuals had higher sleeping scores, fewer mental health issues, and less cardiovascular disease.

**Table 1:** Descriptions of baseline characteristics for the participants and by their metabolic status

	Total (n = 119,424)	MUO (n = 92,195)	MHO (n = 27,229)	P-value
Age, mean (SD), y	56.7 (7.9)	57.9 (7.5)	52.8 (7.8)	< 0.01
Male, n (%)	55,958 (46.9)	45,845 (49.7)	10,113 (37.1)	< 0.01
Body mass index, mean (SD), kg/m^2^	33.9 (3.9)	34.1 (4.0)	33.3 (3.3)	< 0.01
Physical activity, n (%)				
No MVPA	18,225 (15.3)	14,362 (15.6)	3,893 (14.3)	< 0.01
Low PA	26,229 (22.0)	19,871 (21.6)	6,358 (23.4)	< 0.01
Medium PA	14,426 (12.1)	10,996 (11.9)	3,430 (12.6)	< 0.01
High PA	32,982 (27.6)	25,226 (27.4)	7,756 (28.5)	< 0.01
Smoking status, n (%)				
Current smoker	11,659 (9.8)	8,418 (9.1)	3,241 (11.9)	< 0.01
Previous smoker	46,109 (38.6)	37,097 (40.2)	9,012 (33.1)	< 0.01
Never	60,883 (51.0)	46,067 (50.0)	14,816 (54.4)	< 0.01
Alcohol drinking status, n (%)				
Current drinker	106,901 (89.5)	82,320 (89.3)	24,581 (90.3)	< 0.01
Previous drinker	5,461 (4.6)	4,353 (4.7)	1,108 (4.1)	< 0.01
Never	6,689 (5.6)	5,231 (5.7)	1,458 (5.4)	< 0.01
Employment status, n (%)				
Unemployment	9,075 (7.6)	7,366 (8.0)	1,709 (6.3)	< 0.01
Retired	39,431 (33.0)	34,485 (37.4)	4,946 (18.2)	< 0.01
Employed in night shift work	3,170 (2.7)	2,220 (2.4)	950 (3.5)	< 0.01
Employed in day shift work	5,063 (4.2)	3,571 (3.9)	1,492 (5.5)	< 0.01
Employed not in shift work	57,398 (48.1)	40,812 (44.3)	16,586 (60.9)	< 0.01
Income, n (%)				
< £ 18,000	27,955 (23.4)	23,026 (25.0)	4,929 (18.1)	< 0.01
£ 18,000 - £ 30,999	26,288 (22.0)	20,670 (22.4)	5,618 (20.6)	< 0.01
£ 31,000 - £ 51,999	24,859 (20.8)	18,304 (19.9)	6,555 (24.1)	< 0.01
£ 52,000 - £ 100,000	17,086 (14.3)	11,957 (13.0)	5,129 (18.8)	< 0.01
> £ 100,000	3,610 (3.0)	2,521 (2.7)	1,089 (4.0)	< 0.01
Healthy sleep score, n (%)				
0 - 1	7,307 (6.1)	5,869 (6.4)	1,438 (5.3)	< 0.01
2 - 3	61,100 (51.2)	47,683 (51.7)	13,417 (49.3)	< 0.01
4 - 5	51,017 (42.7)	38,643 (41.9)	12,374 (45.4)	< 0.01
TDI, mean (SD)	-0.8 (3.3)	-0.8 (3.3)	-0.8 (3.3)	0.39
Vegetable and fruit intake, mean (SD)	4.6 (3.1)	4.6 (3.0)	4.6 (3.2)	0.8
Sedentary behavior, mean (SD), hour/day	5.6 (2.7)	5.6 (2.7)	5.4 (2.7)	< 0.01
Mental health issue, n (%)	45,200 (37.8)	34,389 (37.3)	10,811 (39.7)	< 0.01
Cardiovascular disease, n (%)	12,077 (10.1)	9,909 (10.7)	2,168 (8.0)	< 0.01
Diabetes, n (%)	16,249 (13.6)	16,249 (17.6)	-*	-*
Hypertension, n (%)	87,339 (73.1)	87,339 (94.7)	-*	-*
Hypercholesterolemia, n (%)	33,865 (28.4)	33,865 (36.7)	-*	-*

MUO, metabolically unhealthy obesity; MHO, metabolically healthy obesity; MVPA, moderate-to-vigorous physical activity; PA, physical activity; TDI, Townsend deprivation index

* Not available

During follow-up, a total of 5,506 incident AF events and 117 AF deaths were observed. There were 16 AF events occurred within the first two weeks after patients received a heart valve surgery, demonstrating that the majority of AF events were not post-operation related. Independent associations of metabolic status and PA with incident AF were demonstrated in **STable 2** (for the age- and sex-adjusted model) and Table 2 (for the fully adjusted model). MHO was significantly associated with a 35% reduced risk of incident AF compared with MUO (HR = 0.65, 95% CI: 0.57–0.73) (Table 2). Compared with no MVPA, medium and high PA were significantly associated with decreased risk of incident AF (HR = 0.84, 95% CI: 0.75–0.95, and HR = 0.88, 95% CI: 0.80–0.97 for medium and high PA, respectively) (Table 2). There was no significant association found for AF mortality. **SFigure 2** shows the independent associations of all other variables with incident AF in the fully adjusted model.

**Table 2:** Independent and mutually adjusted associations of metabolic status and physical activity with atrial fibrillation*

	AF		AF death	
Exposure*	No. of cases/No. of total participants	HR (95% CI), p-value	No. of cases/No. of total participants	HR (95% CI), p-value
***Metabolic status***				
MUO	2,904/54,627	Ref	58/54,627	Ref
MHO	320/16,861	0.65 (0.57, 0.73), p < 0.01	5/16,861	0.67 (0.27, 1.70), p = 0.40
***Physical activity***				
No MVPA	715/13,600	Ref	18/13,600	Ref
low	887/20,727	0.91 (0.83, 1.01), p = 0.07	17/20,727	0.76 (0.39, 1.49), p = 0.43
medium	456/11,317	0.84 (0.75, 0.95), p < 0.01	8/11,317	0.62 (0.27, 1.44), p = 0.26
high	1,166/25,844	0.88 (0.80, 0.97), p = 0.01	20/25,844	0.62 (0.32, 1.20), p = 0.16

* ‘Metabolically healthy’ indicated none of the metabolic disorders, and ‘metabolically unhealthy’ indicated at least one of the metabolic disorders, where metabolic disorders included hypertension, hypercholesterolemia, and diabetes. Participants who met at least one of the following criteria were deemed to be ***hypertensive***: systolic blood pressure ≥ 140 mmHg, diastolic blood pressure ≥ 90 mmHg, taking anti-hypertensive medications, a self-reported history of hypertension or ICD-10 code I10-I15 and ICD-9 code 401 and 403. Self-reported history of high cholesterol, taking medications, or ICD-10 code E780 and ICD-9 code 2720 was the criteria for ***hypercholesterolemia***. ***Diabetes mellitus*** was defined as hospital records of diabetes at baseline based on ICD-10 code E10-E14 and ICD-9 code 250 or taking medications or a self-reported history of type 1 or type 2 diabetes. In line with public health guidelines, PA was categorized into four groups according to the level of moderate-to-vigorous PA (MVPA): none (0 MET-mins per week for MVPA), low (< 600 MET-mins per week), medium (600 - 1200 MET-mins per week), and high (≥ 1200 MET-mins per week)

* Models adjusted for age, sex, BMI, smoking status, alcohol drinking status, income, sleep scores, mental health issues, employment status, Townsend deprivation index, vegetable and fruit intake, sedentary behavior, cardiovascular disease and mutually adjusted for physical activity or metabolic status as appropriate

AF, atrial fibrillation; MUO, metabolically unhealthy obesity; MHO, metabolically healthy obesity; MVPA, moderate-to-vigorous physical activity; HR, hazard ratio; CI, confidence interval

**Fig. 1:**
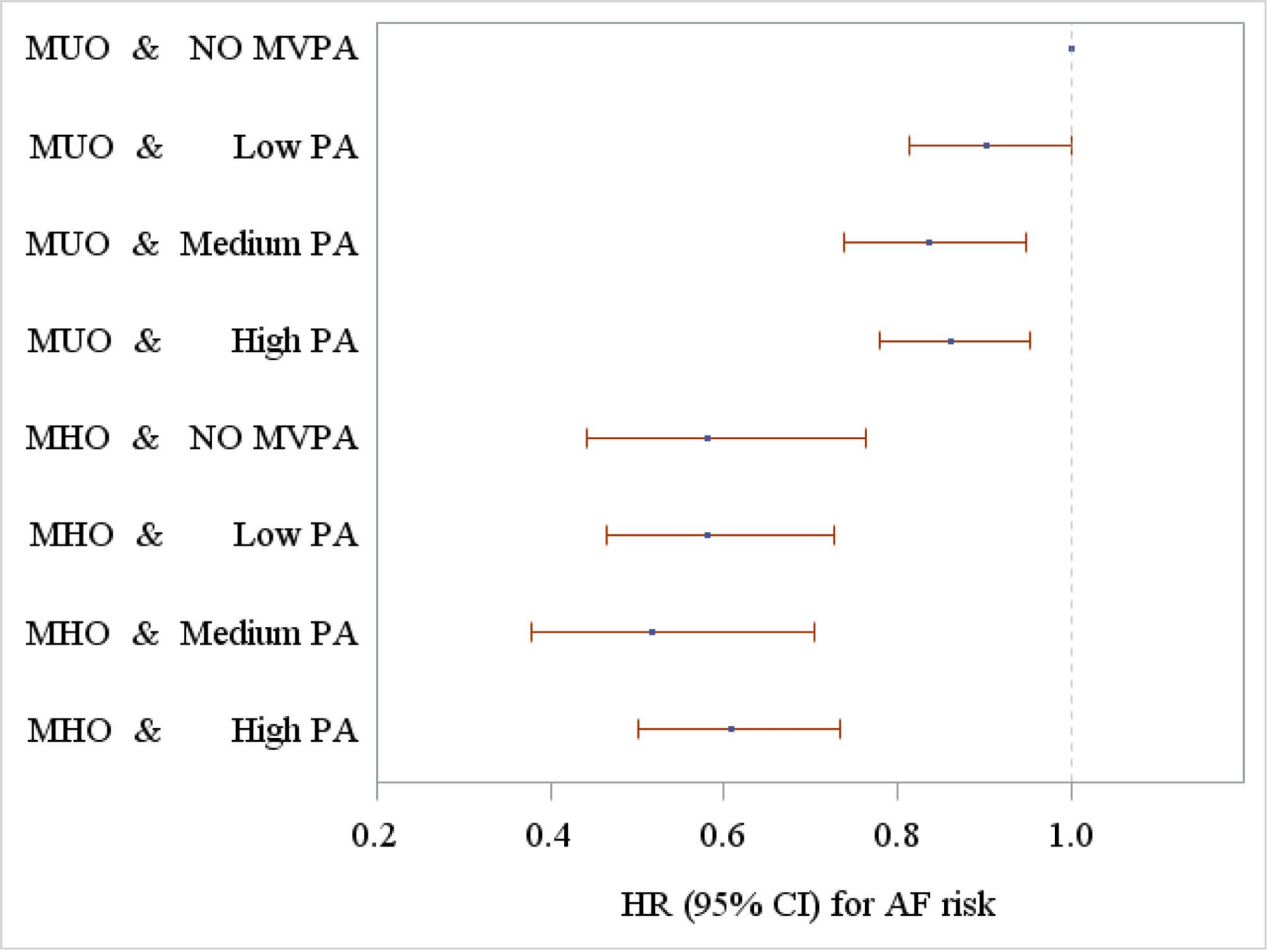
The joint associations of metabolic status and physical activity with incident atrial fibrillation risk (note- MUO, metabolically unhealthy obesity; MHO, metabolically healthy obesity; MVPA, moderate-to-vigorous physical activity; PA, physical activity; HR, hazard ratio; CI, confidence interval

Figure 1 illustrates the joint associations of metabolic status and PA with AF risk in the obese participants, showing the HRs and 95% CIs of different groups defined by metabolic status and PA, taking the MUO and no MVPA as reference. The number of AF events in different groups and the joint associations in different groups defined by metabolic status and physical activity were presented in **STable 3**. Among the MUO participants, individuals with medium and high PA had significantly lower risk of incident AF compared with no MVPA (HR = 0.84, 95% CI: 0.74–0.95, and HR = 0.87, 95% CI: 0.78–0.96 for medium and high PA, respectively) (**STable 3**); however the modification effects of the three PA groups when compared with no MVPA did not significantly differ (p = 0.40). No significant modification of PA was found on the AF risk among individuals with MHO (p = 0.88).

**STable 4** and Table 3 respectively show the independent and joint associations of metabolic status and PA with risk of AF in different subgroups by age and sex. Among participants with MUO, the modification of PA on AF risk was significantly higher in females than that in males (p < 0.01); however there was no significant modification of PA in different age groups (p = 0.85).

**Table 3:** Subgroup analyses for the joint associations of metabolic status and physical activity with incident atrial fibrillation risk*

	Sex				Age			
	**Male**		**Female**		**< 65 years**		**≥ 65 years**	
**Metabolic status & PA**	No. of cases/No. of total participants	HR (95% CI), p-value	No. of cases/No. of total participants	HR (95% CI), p-value	No. of cases/No. of total participants	HR (95% CI), p-value	No. of cases/No. of total participants	HR (95% CI), p-value
MUO & No MVPA	439/5,643	Ref	219/5,021	Ref	415/8,668	Ref	243/1,996	Ref
MUO & Low PA	569/8,133	0.97 (0.86, 1.10), p = 0.64	232/7,561	0.78 (0.65, 0.94), p < 0.01	494/12,834	0.89 (0.78, 1.01), p = 0.08	307/2,860	0.92 (0.78, 1.09), p = 0.34
MUO & Medium PA	294/4,481	0.90 (0.78, 1.05), p = 0.18	118/4,120	0.70 (0.56, 0.88), p < 0.01	255/6,875	0.85 (0.72, 0.99), p = 0.04	157/1,726	0.77 (0.63, 0.95), p = 0.01
MUO & High PA	721/11,628	0.85 (0.75, 0.96), p = 0.01	312/8,040	0.89 (0.75, 1.07), p = 0.21	633/15,245	0.89 (0.79, 1.02), p = 0.08	400/4,423	0.77 (0.65, 0.91), p < 0.01
MHO & No MVPA	29/1,079	0.56 (0.39, 0.82), p < 0.01	28/1,857	0.62 (0.42, 0.92), p = 0.02	48/2,777	0.50 (0.37, 0.67), p < 0.01	9/159	0.52 (0.27 1.01), p = 0.05
MHO & Low PA	48/1,785	0.59 (0.44, 0.80), p < 0.01	38/3,248	0.52 (0.37, 0.74), p < 0.01	65/4,710	0.44 (0.34, 0.57), p < 0.01	21/323	0.64 (0.41, 1.00), p = 0.05
MHO & Medium PA	23/1,034	0.52 (0.34, 0.79), p < 0.01	21/1,682	0.53 (0.34, 0.83), p < 0.01	36/2,522	0.45 (0.32, 0.63), p < 0.01	8/194	0.40 (0.20, 0.81), p = 0.01
MHO & High PA	84/2,878	0.63 (0.50, 0.80), p < 0.01	49/3,298	0.56 (0.41, 0.77), p < 0.01	99/5,651	0.49(0.39, 0.61), p < 0.01	34/525	0.59 (0.41, 0.84), p < 0.01

*Models adjusted for age, sex, body mass index, smoking status, alcohol drinking status, income, sleep scores, mental health issues, employment status, Townsend deprivation index, vegetable and fruit intake, sedentary behavior, and cardiovascular disease

MUO, metabolically unhealthy obesity; MHO, metabolically healthy obesity; MVPA, moderate-to-vigorous physical activity; PA, physical activity; HR, hazard ratio; CI, confidence interval

**Fig. 2:**
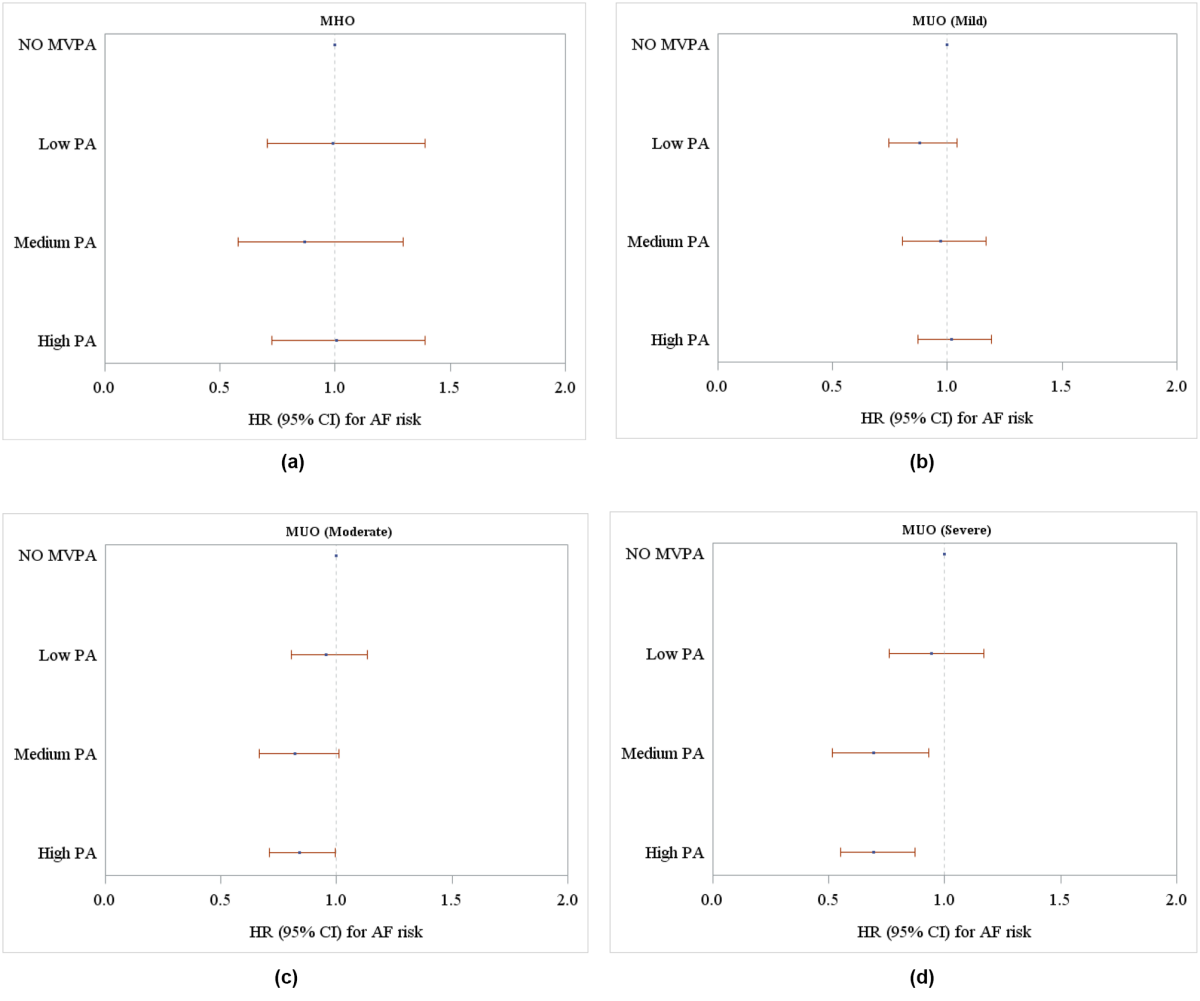
Associations between physical activity and incident atrial fibrillation risk stratified by different metabolic status: a for MHO; b for mild MUO, c for moderate MUO, d for severe MUO. (note- Metabolically unhealthy status was categorized into: mild (with only one of the metabolic disorders), moderate (with two of the metabolic disorders), severe (with all of three metabolic disorders). Models were adjusted for age, sex, body mass index, smoking status, alcohol drinking status, income, sleep scores, mental health issues, employment status, Townsend deprivation index, vegetable and fruit intake, sedentary behavior, and cardiovascular disease. MUO, metabolically unhealthy obesity; MHO, metabolically healthy obesity; MVPA, moderate-to-vigorous physical activity; PA, physical activity; HR, hazard ratio; CI, confidence interval)

In the exploratory analyses, there was a significant modification of PA on the risk of AF among the participants with severe MUO: HR = 0.70, 95% CI: 0.52–0.94 and HR = 0.70, 95% CI: 0.55–0.88 for medium and high PA when compared with no MVPA (Fig. 2d and **STable 5**). As the severity of MUO increased, the modification of PA on incident AF risk was elevated accordingly (p for trend < 0.01; **STable 5**).

Non-significantly additive or multiplicative modification was found in the whole obese population, as shown in **STable 6**. The sensitivity analyses yielded similar results to the main findings (**STable 7–9**).

## Discussion

In this study, our principal findings were as follows: (i) among obese participants, both metabolically healthy status and PA (medium and high PA) were significantly associated with reduced risk of incident AF as expected compared with MUO and no MVPA, respectively; (ii) individuals with medium and high PA had a lower risk of incident AF, especially for the medium PA; (iii) among the MUO participants, individuals with medium and high PA had significantly lower risk of AF compared with no MVPA, while there was no significant risk modification by PA on AF risk among individuals with MHO; and (iv) with worsening of MUO (mild, moderate, and severe), the risk reduction of PA on AF risk was elevated accordingly.

There were 77% of participants with MUO in our study (Table 1), broadly similar to the study by Cao et al. (73%) [32]. A nationwide study conducted in Korea with an average follow-up of 7.5 years reported AF incidence rates for MHO and MUO groups (1.10 and 2.88 per 1,000 person-years respectively), and also showed the adjusted HR for MHO and MUO groups (1.30, 95% CI: 1.14–1.48; 1.71, 95% CI: 1.56–1.86, respectively) when compared with metabolically healthy non-obese group [28]. Their results suggested that individuals with MUO had a higher risk of incident AF than the remaining obese participants, consistent with our study. However, the relationship between MUO and AF has been debated. Another study conducted in Norway presented the similar AF risks for the MHO and MUO groups (adjusted HR = 1.6, 95% CI: 1.2–2.1; adjusted HR = 1.6, 95% CI: 1.3–1.9, respectively) compared with the metabolically healthy non-obese group, while the crude HRs showed a lower AF risk for MUO group (crude HR = 1.7, 95% CI: 1.3–2.1) than that for the MHO group (crude HR = 2.4, 95% CI: 2.1–2.8) [29]. Nevertheless, the majority of previous studies regarded the metabolically healthy non-obese individuals as the reference group, rather than the MHO participants. In this study, we investigated AF risk in the MUO group compared with that in the MHO counterpart. Individuals with MUO have impaired vascular function, inflammation and fibrosis at the cellular level, as well as remodeling of the left atrium, left ventricle, and arterial wall, which eventually results in a high risk of developing AF [33–36].

Previous studies have reported that the individuals participating in any MVPA could result in a lower risk of AF [17, 19]. Furthermore, Garnvik et al. found that higher levels of PA were associated with lower AF risk among obese individuals [37]. In the present study, we extended these observations showing that among the MUO participants, individuals with medium and high PA had significantly lower risk of AF, especially the medium PA, while no significant modification of PA was found on AF risk among individuals with MHO. One possible explanation was that PA can strengthen the ability of physiological adjustments including blood flow and vascular function regulation in obesity, which may modify the relationship between metabolic status and AF risk [38]. One study by Lee et al. showed that regardless of the type of metabolic disorder, the metabolic unhealthy status was severer with an increasing number of metabolic disorders [12]. Besides, our study found that with more severe MUO (mild, moderate, and severe), the risk reduction of PA on incident AF risk was elevated accordingly. Less than 40% of obese participants achieved medium or high PA (Table 1). Indeed, obese individuals rarely exercise due to the various complications of obesity including decreased inspiratory muscle strength and restrictive ventilation [39, 40]. However, MVPA can strengthen the ability of physiological adjustments, reduced the risk of cardiovascular disease, and enhance the quality of life [41]. In our analysis, we found the medium PA, but not high PA, had significantly higher modification on the AF risk compared with no MVPA among individuals with MUO. Sanchis-Gomar et al. demonstrated that high level of PA can increase arterial pressure, and stretch of the atrial wall, followed by micro-trauma, inflammation, and fibrosis, which may result in arrhythmogenic and reduce modification effect of PA on the AF risk in participants with MUO [42]. Nevertheless, the modification of medium PA was not significantly higher than low or high PA on AF risk in MUO. One possible reason may be the small sample size or a potential absence of the modification. Therefore, our results should be interpreted with care and caution, requiring further research to validate this relationship.

Through subgroup analysis, among participants with MUO, the modification of PA on AF risk was significantly higher in females than that in males. Wan et al. showed that females reporting PA had lower AF incidence [43]. One possible mechanism was that the appropriate level of PA for males was higher than for females, while a high level of PA may decrease the modification of PA on the AF risk among MUO participants [42–44]. By contrast, we found no significant impact of PA on different age groups among MUO, which may indicate that the PA could modify the AF risk to a similar level across different age strata. Nevertheless, more research is needed to further validate these subgroup findings with an exploratory nature.

### Strengths and limitations

This study has some strengths. We categorized the MUO into three groups to further explore the impact of PA on the relationship between MUO and incident AF risk. To our best knowledge, this study is the first attempt to investigate the joint association of metabolic status and PA with AF risk among obese participants. Similar results from sensitivity analyses further supported the robustness of the main findings. While the current guidelines consistently emphasized the importance of AF identification and prevention [20, 45], our study results suggested that participants reporting medium PA were related with lowest AF risk. This may provide some evidence-based data to PA recommendation for the AF prevention specifically in the obese population, regardless of their metabolic status and their severity of MUO.

Several limitations need to be noted. The AF was diagnosed by trained physicians from different hospitals, which may lead to misdiagnosis. Moreover, our results should be interpreted with caution due to possible bias or residual confounding effects that could not be fully precluded in an observational study design. Although we have taken C-reactive protein and white blood cell counts into consideration in the sensitivity analysis, no other variables related to inflammatory status could be assessed due to unavailability of these data. Moreover, left atrial remodeling and left atrial function could be modified by both exercise and metabolic status, which would therefore influence the relationship between risk of AF and metabolic status and PA. Nevertheless, no data on left atrial remodeling and function were available in our current analysis, thereby weakening the strength of our results to an unknown extent. Furthermore, the information on PA was collected by self-report questionnaire, possibly incurring information bias and overestimation of PA due to social desirability. Taking the low response rate at baseline (5.5%) into consideration, the generalizability of our study findings may be compromised [46]. Additionally, in the exploratory analyses we only assessed the severity of MUO based on the number of metabolic disorders at baseline, regardless of the disease severity and management of the individual MUO components, because there was no sufficient information on evaluating these two aspects. Therefore, solely depending on the number of metabolic disorders may not precisely reflect the granularity for the severity of MUO. Moreover, no analyses were performed to explore the changes in or trajectories of metabolic status and PA in relation to risk of AF, due to the limited data available from the study.

## Conclusion

MHO was significantly associated with a reduced risk of incident AF compared with MUO in obese participants. PA could significantly modify the relationship between metabolic status and risk of AF among MUO participants, with particular benefits of PA associated with the reduced AF risk as the MUO severity elevated.

## Electronic supplementary material

Below is the link to the electronic supplementary material.

Supplementary Material 1

Supplementary Material 2

## Data Availability

The data can be available on application to the UK Biobank (www.ukbiobank.ac.uk/).

## Abbreviations

AF: Atrial fibrillation.
MHO: Metabolically healthy obesity.
PA: Physical activity.
MVPA: Moderate-to-vigorous physical activity.
TDI: Townsend deprivation index.
HR: Hazard ratio.
CI: Confidence interval.
