# Supplementary material for: Association between metabolically healthy obesity and risk of atrial fibrillation: taking physical activity into consideration
Source: Cardiovasc Diabetol. 2022 Oct 13;21:208. doi: 10.1186/s12933-022-01644-z (PMC9563485; doi:10.1186/s12933-022-01644-z)

Supplemental Contents

STable 1. Identification of baseline and incident atrial fibrillation in this study 2

STable 2. Independent associations of metabolic status and physical activity with atrial fibrillation from the model adjusted for age and sex 3

STable 3. Number of atrial fibrillation events and the joint associations in different groups defined by metabolic status and physical activity 4

STable 4. Subgroup analyses for the independent and mutually adjusted associations of metabolic status and physical activity with incident atrial fibrillation risk* 5

STable 5. Associations between physical activity and incident atrial fibrillation risk stratified by different metabolic status* 7

STable 6. Effect modification of metabolic status and physical activity with incident atrial fibrillation risk where metabolic status is the exposure of interest and physical activity is the potential effect modifier* 8

STable 7. Joint associations of metabolic status and physical activity with incident atrial fibrillation risk using multiple imputation technique for missing data*.................................... 9

STable 8. Joint associations of metabolic status and physical activity with incident atrial fibrillation risk using different definition of metabolic status* 10

STable 9. Joint associations of metabolic status and physical activity with incident atrial fibrillation risk by further adjusted for other variables*... 11

SFigure 1. Flow diagram showing participant selection for this study 12

SFigure 2. Independent associations of all other variables with incident atrial fibrillation risk from the fully adjusted model.. 13

STable 1. Identification of baseline and incident atrial fibrillation in this study

Data fields	Fields' names	Data code
20002[1]	Non-cancer illness codes, self-reported	1471, 1483
41270[2]	Diagnoses - ICD10	I48, I480, I481, I482, I483, I484, I489
41271[2]	Diagnoses – ICD9	4273
40001[3]	Underlying (primary) cause of death: ICD10	I48, I480, I481, I482, I483, I484, I489
40002[3]	Contributory (secondary) causes of death: ICD10	I48, I480, I481, I482, I483, I484, I489
20004[1]	Operation, self-reported	1524, 1553
41272[2]	Operative procedure codes	K223, K571, K575, K621, K622, K623, K624, K625, X501, X502

ICD = international classification of diseases

[1] for baseline AF identification only

[2] for both baseline and incident AF identification

[3] for both incident AF and AF mortality identification

STable 2. Independent associations of metabolic status and physical activity with atrial fibrillation from the model adjusted for age and sex

	**AF**	**AF death**
**Exposure**	HR (95% CI), p-value	HR (95% CI), p-value
Metabolic status		
MUO	Ref	Ref
MHO	0.59 (0.54, 0.65), p < 0.01	0.29 (0.12, 0.71), p < 0.01
Physical activity		
No MVPA	Ref	Ref
low	0.84 (0.77, 0.92), p < 0.01	0.65 (0.37, 1.16), p = 0.15
medium	0.78 (0.70, 0.86), p < 0.01	0.65 (0.33, 1.27), p = 0.21
high	0.79 (0.73, 0.86), p < 0.01	0.55 (0.32, 0.94), p = 0.03

AF, atrial fibrillation; MUO, metabolically unhealthy obesity; MHO, metabolically healthy obesity; MVPA, moderate-to-vigorous physical activity; HR, hazard ratio; CI, confidence interval.

STable 3. Number of atrial fibrillation events and the joint associations in different groups defined by metabolic status and physical activity

**Metabolic status, physical activity**	No. With/Without Events	HR (95% CI), p-value
MUO, no MVPA (n = 14,377)	658/10,664	Ref
MUO, low PA (n = 19,894)	801/15,694	0.91 (0.82, 1.01), p = 0.07
MUO, medium PA (n = 11,009)	412/8,601	0.84 (0.74, 0.95), p < 0.01
MUO, high PA (n = 25,253)	1,033/19,668	0.87 (0.78, 0.96), p < 0.01
MHO, no MVPA (n = 3,894)	57/2,936	0.59 (0.45, 0.77), p < 0.01
MHO, low PA (n = 6,366)	86/5,033	0.56 (0.45, 0.70), p < 0.01
MHO, medium PA (n = 3,430)	44/2,716	0.53 (0.39, 0.72), p < 0.01
MHO, high PA (n = 7,763)	133/6,176	0.61 (0.51, 0.74), p < 0.01
Overall (n = 91,986)	3,224/71,488	-

MUO, metabolically unhealthy obesity; MHO, metabolically healthy obesity; MVPA, moderate-to-vigorous physical activity.

STable 4. Subgroup analyses for the independent and mutually adjusted associations of metabolic status and physical activity with incident atrial fibrillation risk*

	**Sex**				**Age**			
	**Male**		**Female**		**< 65 years**		**≥ 65 years**	
	No. of cases/No. of total participants	HR (95% CI), p-value	No. of cases/No. of total participants	HR (95% CI), p-value	No. of cases/No. of total participants	HR (95% CI), p-value	No. of cases/No. of total participants	HR (95% CI), p-value
Metabolic status								
MUO	2,023/29,885	Ref	881/24,742	Ref	1,797/43,622	Ref	1,107/11,005	Ref
MHO	184/6,776	0.65 (0.56, 0.76), p < 0.01	136/10,085	0.66 (0.55, 0.79), p < 0.01	248/15,660	0.52 (0.45, 0. 60), p < 0.01	72/1,201	0.67 (0.52, 0.85), p < 0.01
Physical activity								
No MVPA	468/6,722	Ref	247/6,878	Ref	463/11,445	Ref	252/2,155	Ref
Low	617/9,918	0.98 (0.86, 1.10), p = 0.69	270/10,809	0.79 (0.66, 0.94), p < 0.01	559/17,544	0.89 (0.78, 1.00), p = 0.06	328/3,183	0.93 (0.79, 1.10), p = 0.40
Medium	317/5,515	0.90 (0.78, 1.04), p = 0.16	139/5,802	0.72 (0.59, 0.89), p < 0.01	291/9,397	0.85 (0.74, 0.99), p = 0.04	165/1,920	0.77 (0.63, 0.94), p < 0.01

High	805/14,506	0.87 (0.78, 0.98), $p = 0.02$	361/11,338	0.89 (0.76, 1.06), $p = 0.18$	732/20,896	0.90 (0.80, 1.02), $p = 0.10$	434/4,948	0.79 (0.67, 0.92), $p < 0.01$

MUO, metabolically unhealthy obesity; MHO, metabolically healthy obesity; MVPA, moderate-to-vigorous physical activity; HR, hazard ratio; CI, confidence interval.

* Models adjusted for age, sex, body mass index, smoking status, alcohol drinking status, income, sleep scores, mental health issues, employment status, socioeconomic status (TDI: Townsend deprivation index), vegetable and fruit intake, sedentary behavior, cardiovascular disease and mutually adjusted for physical activity or metabolic status as appropriate.

STable 5. Associations between physical activity and incident atrial fibrillation risk stratified by different metabolic status*

Physical activity	MHO	MUO		
		Mild	Moderate	Severe
No MVPA	Ref	Ref	Ref	Ref
Low	0.95 (0.68, 1.33), p = 0.76	0.90 (0.76, 1.07), p = 0.23	0.96 (0.81, 1.14), p = 0.63	0.93 (0.75, 1.16), p = 0.52
Medium	0.88 (0.59, 1.31), p = 0.53	0.97 (0.80, 1.17), p = 0.73	0.83 (0.67, 1.02), p = 0.07	0.70 (0.52, 0.94), p = 0.02
High	1.01 (0.73, 1.39), p = 0.98	1.04 (0.89, 1.21), p = 0.65	0.84 (0.71, 1.00), p = 0.04	0.70 (0.55, 0.88), p < 0.01

AF, atrial fibrillation; MUO, metabolically unhealthy obesity; MHO, metabolically healthy obesity; MVPA, moderate-to-vigorous physical activity; PA, physical activity; HR, hazard ratio; CI, confidence interval.

* Metabolically unhealthy status were categorized into: ***Mild*** (only one of the metabolic disorders), ***moderate*** (two of the metabolic disorders), ***severe*** (all of the metabolic disorders). Metabolic disorders: hypertension, hypercholesterolemia, and diabetes.

Models were adjusted for age, sex, body mass index, smoking status, alcohol drinking status, income, sleep scores, mental health issues, employment status, socioeconomic status (TDI: Townsend deprivation index), vegetable and fruit intake, sedentary behavior, and cardiovascular disease.

STable 6. Effect modification of metabolic status and physical activity with incident atrial fibrillation risk where metabolic status is the exposure of interest and physical activity is the potential effect modifier*

	**MHO**		**MUO**		HR (95% CI) for MUO within Strata of PA
	No. of cases/No. of total participants	HR (95% CI), p-value	No. of cases/No. of total participants	HR (95% CI), p-value	
Any MVPA	658/10,664	Ref	2,246/43,963	1.52 (1.33, 1.73), p < 0.01	1.50 (1.31, 1.71), p < 0.01
No MVPA	57/2,936	1.02 (0.76, 1.36), p = 0.91	263/13,925	1.73 (1.49, 2.00), p < 0.01	1.72 (1.31, 2.27), p < 0.01

MHO, metabolically healthy obesity; MUO, metabolically unhealthy obesity; PA, physical activity; MVPA, moderate-to-vigorous physical activity; HR, hazard ratio; CI, confidence interval.
* Measure of effect modification on additive scale: RERI (95% CI) = 0.20 (-0.12, 0.51), AP (95% CI) = 0.11 (-0.18, 0.30), and S = 1.37 (0.74, 2.53).
Measure of effect modification on multiplicative scale: ratio of HRs (95% CI) = 1.12 (0.65, 1.59)
Models were adjusted for age, sex, body mass index, smoking status, alcohol drinking status, income, sleep scores, mental health issues, employment status, Townsend deprivation index, vegetable and fruit intake, sedentary behavior, and cardiovascular disease.

STable 7. Joint associations of metabolic status and physical activity with incident atrial fibrillation risk using multiple imputation technique for missing data*

**Metabolic status & Physical activity**	HR (95% CI), p-value
MUO & No MVPA	Ref
MUO & Low PA	0.93 (0.85, 1.02), p = 0.13
MUO & Medium PA	0.89 (0.79, 0.99), p = 0.04
MUO & High PA	0.90 (0.82, 0.99), p = 0.03
MHO & No MVPA	0.60 (0.47, 0.77), p < 0.01
MHO & Low PA	0.59 (0.48, 0.73), p < 0.01
MHO & Medium PA	0.59 (0.45, 0.77), p < 0.01
MHO & High PA	0.64 (0.54, 0.76), p < 0.01

MUO, metabolically unhealthy obesity; MHO, metabolically healthy obesity; MVPA, moderate-to-vigorous physical activity; PA, physical activity; HR, hazard ratio; CI, confidence interval.
* Models were adjusted for age, sex, body mass index, smoking status, alcohol drinking status, income, sleep scores, mental health issues, employment status, socioeconomic status (TDI: Townsend deprivation index), vegetable and fruit intake, sedentary behavior, and cardiovascular disease.

STable 8. Joint associations of metabolic status and physical activity with incident atrial fibrillation risk using different definition of metabolic status*

**Metabolic status & Physical activity**	HR (95% CI), p-value
MUO & No MVPA	Ref
MUO & Low PA	0.93 (0.83, 1.04), p = 0.19
MUO & Medium PA	0.86 (0.75, 0.98), p = 0.02
MUO & High PA	0.86 (0.77, 0.96), p < 0.01
MHO & No MVPA	0.91 (0.75, 1.11), p = 0.36
MHO & Low PA	0.77 (0.65, 0.91), p < 0.01
MHO & Medium PA	0.71 (0.57, 0.89), p < 0.01
MHO & High PA	0.86 (0.74, 0.99), p = 0.03

MUO, metabolically unhealthy obesity; MHO, metabolically healthy obesity; MVPA, moderate-to-vigorous physical activity; PA, physical activity; HR, hazard ratio; CI, confidence interval.
* Models were adjusted for age, sex, body mass index, smoking status, alcohol drinking status, income, sleep scores, mental health issues, employment status, socioeconomic status (TDI: Townsend deprivation index), vegetable and fruit intake, sedentary behavior, and cardiovascular disease.

STable 9. Joint associations of metabolic status and physical activity with incident atrial fibrillation risk by further adjusted for other variables*

**Metabolic status & Physical activity**	HR (95% CI), p-value
MUO & No MVPA	Ref
MUO & Low PA	0.88 (0.79, 0.98), p = 0.02
MUO & Medium PA	0.81 (0.71, 0.92), p < 0.01
MUO & High PA	0.82 (0.74, 0.91), p < 0.01
MHO & No MVPA	0.55 (0.41, 0.73), p < 0.01
MHO & Low PA	0.46 (0.36, 0.59), p < 0.01
MHO & Medium PA	0.48 (0.35, 0.66), p < 0.01
MHO & High PA	0.56 (0.46, 0.68), p < 0.01

MUO, metabolically unhealthy obesity; MHO, metabolically healthy obesity; MVPA, moderate-to-vigorous physical activity; PA, physical activity; HR, hazard ratio; CI, confidence interval.
* Models were adjusted for age, sex, height, C-reactive protein, white blood cell counts, smoking status, alcohol drinking status, income, sleep scores, mental health issues, employment status, socioeconomic status (TDI: Townsend deprivation index), vegetable and fruit intake, sedentary behavior, and cardiovascular disease.

SFigure 1. Flow diagram showing participant selection for this study

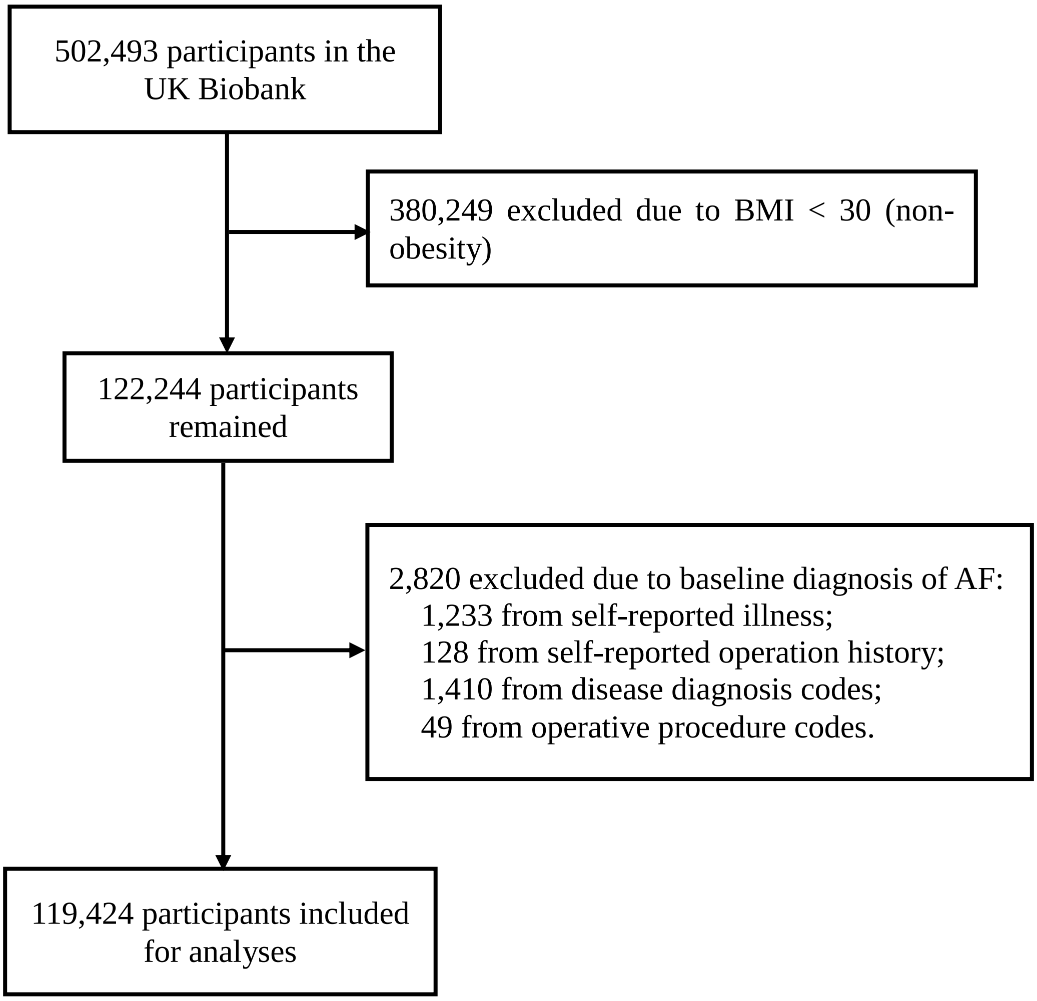

SFigure 2. Independent associations of all other variables with incident atrial fibrillation risk from the fully adjusted model

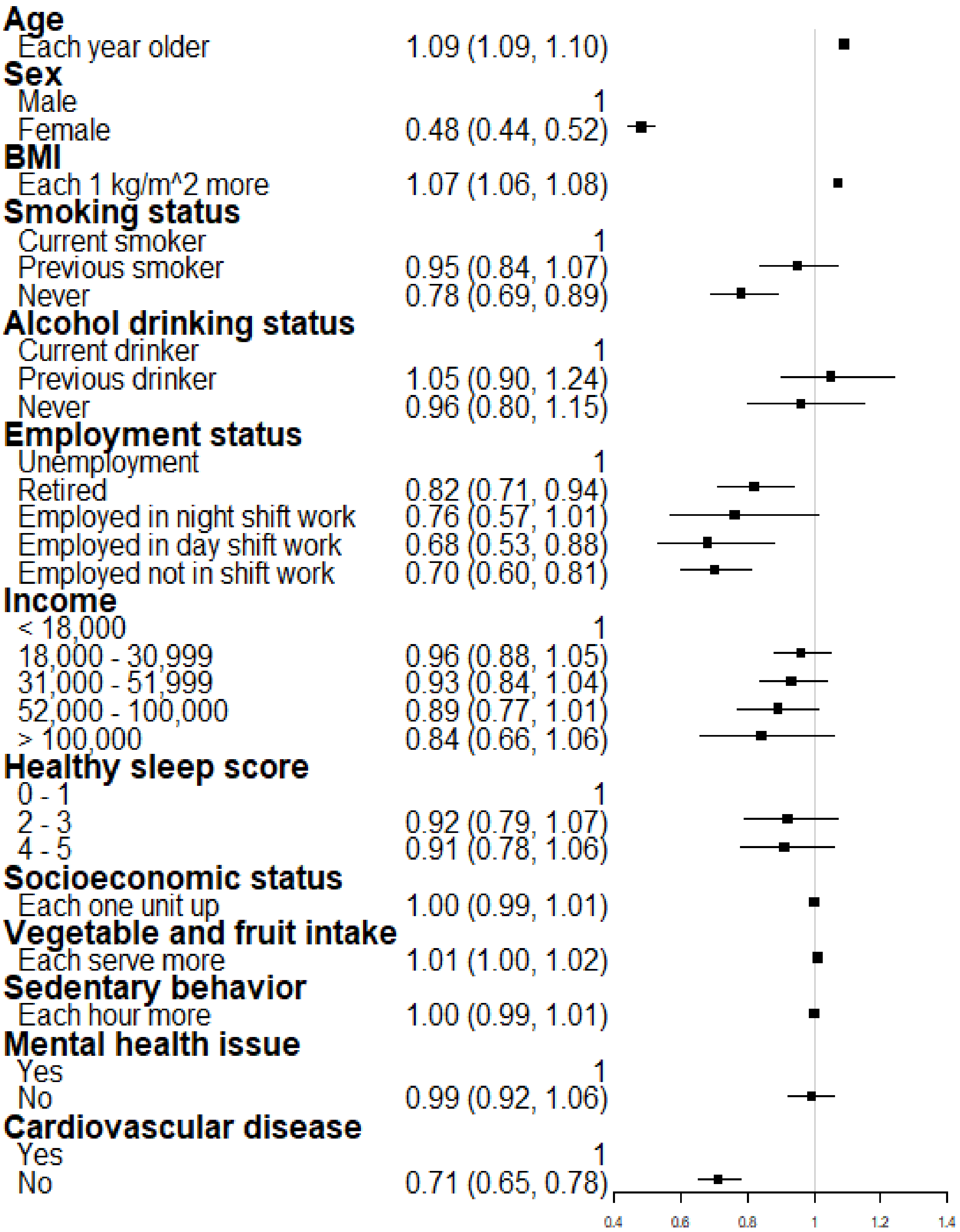

Supplement: Supplementary file 1 — Supplementary Material 1 [file 12933_2022_1644_MOESM1_ESM.pdf]
